# A Phase I study of pevonedistat plus capecitabine plus oxaliplatin in patients with advanced gastric cancer refractory to platinum (NCCH-1811)

**DOI:** 10.2144/fsoa-2021-0023

**Published:** 2021-05-28

**Authors:** Hirokazu Shoji, Daisuke Takahari, Hiroki Hara, Kengo Nagashima, Jun Adachi, Narikazu Boku

**Affiliations:** 1 Department of Gastrointestinal Medical Oncology, National Cancer Center Hospital, 5-1-1 Tsukiji, Chuo-ku, Tokyo, 104-0045, Japan; 2 Department of Gastroenterology, Cancer Institute Hospital of Japanese Foundation for Cancer Research, 3-8-31 Ariake, Koto-ku, Tokyo, 135-550, Japan; 3 Department of Gastroenterology, Saitama Cancer Center, 780 Komuro, Inamachi, Kitaadachi-gun, Saitama, 362-0806, Japan; 4 Biostatistics Unit, Clinical and Translational Research Center, Keio University Hospital, 35 Shinanomachi, Shinjuku-ku, Tokyo, 160-8582, Japan; 5 Laboratory of Clinical & Analytical Chemistry, National Institute of Biomedical Innovation, Health & Nutrition, Ibaraki, Osaka, 567-0085, Japan; 6 Laboratory of Proteomics for Drug Discovery, Center for Drug Design Research, National Institute of Biomedical Innovation, Health & Nutrition, Ibaraki, Osaka, 567-0085, Japan

**Keywords:** capecitabine, gastric cancer, oxaliplatin, pevonedistat, salvage line

## Abstract

Based on synergistic anti-tumor effects between blockades of NEDD8 activating enzyme and a platinum in preclinical studies, this Phase I study is designed to investigate the safety and tolerability of pevonedistat in combination with capecitabine plus oxaliplatin as third-line or later treatment in patients with unresectable advanced/recurrent gastric cancer who were previously treated with fluoropyrimidines and platinum (cisplatin or oxaliplatin) as the first-line treatment and paclitaxel (including nab-paclitaxel) as the second-line treatment. The aim of this trial is to determine the recommended dose of pevonedistat and to see its pharmacokinetics in combination with capecitabine plus oxaliplatin in the dose-finding part and explore its efficacy and safety in the expansion part.

**Trial registration number:** jRCT2031190020 (jRCTs: the Japan Registry of Clinical Trials).

## Lay abstract

A Phase I study of pevonedistat plus capecitabine plus oxaliplatin (CapeOX) (NCCH-1811) is designed to investigate the safety and tolerability of pevonedistat in combination with CapeOX as third-line or later treatment in patients with unresectable advanced/recurrent gastric cancer who were previously treated with fluoropyrimidines and platinum (cisplatin or oxaliplatin) as the first-line treatment and paclitaxel (including nab-paclitaxel) as the second-line treatment. The aim is to determine the recommended dose of pevonedistat and to see its pharmacokinetics in combination with CapeOX in the dose finding part and explore its efficacy and safety in the expansion part.

## Background & rationale

Gastric cancer is the fourth most common cancer worldwide, with approximately 18,079,000 new cases diagnosed annually. It is the third leading cause of cancer-related death, with approximately 9,555,000 deaths annually [1]. In Japan, gastric cancer is the second most common malignant disease in men and the fourth most common in women. While the age adjusted mortality rate of gastric cancer has been continuously decreasing of late, it still shows the third highest mortality rate [2]. Chemotherapy is the standard treatment for advanced unresectable or recurrent gastric cancer (AGC) patients to obtain palliation of symptoms and improve survival. However, the prognosis of patients with AGC remains poor with median survival times about 1 year [3,4].

Currently, the standard chemotherapy as first-line treatment for AGC is doublet chemotherapy with fluoropyrimidine and platinum (trastuzumab is added for human epidermal growth factor receptor 2 [HER2]-positive patients) [5]. Oral fluoropyrimidines (e.g., capecitabine or S-1) and oxaliplatin are preferred to infusions of 5-fluorouracil and cisplatin, respectively, because of their noninferior efficacy, convenience and better tolerance [4,6–8]. As second-line treatment, the standard chemotherapy is combination of paclitaxel and ramucirumab based on the result of the RAINBOW trial [9], which demonstrated the superiority of ramucirumab-paclitaxel combination to paclitaxel alone; median survival time (MST) of 9.6 versus 7.4 months (hazard ratio [HR] = 0.807; p = 0.017), respectively.

As third- or later line treatment, monotherapy with irinotecan is recommended in the Japanese Gastric Treatment Guideline, based on the WJOG 4007 trial showing that the proportion of patients who received third-line chemotherapy after failure in the second-line treatment was as high as 89% and 71% in the paclitaxel- and irinotecan-treated groups, respectively, and overall survival was favorable in both groups [10]. The ATTRACTION-2 trial which was a multicenter, double-blinded, randomized trial comparing nivolumab, an anti-PD-1 antibody, with placebo in AGC patients after failure of two or more chemotherapy regimens showed that nivolumab prolonged overall survival compared with placebo (MST: 5.32 vs 4.14 months; HR = 0.63; p < 0.0001) [11]. In Japan, nivolumab was approved for the treatment of AGC in September 2017, and it has been recognized as one of the standard chemotherapies for third- or later line treatment. In addition, a randomized, double-blind, Phase III trial (TAGS) showed overall survival advantage of trifluridine/tipiracil over placebo in patients with AGC who were intolerant, or refractory to two or more standard chemotherapy regimens [12]. The MST was 5.7 months in the trifluridine/tipiracil group versus 3.6 months in the placebo group (HR: 0.69; p = 0.00029). Based on these data, trifluridine/tipiraci was also approved for the treatment of AGC in August 2019 in Japan. Meanwhile, trastuzumab deruxtecan was approved in Japan in September 2020 for HER2-positive AGC patients [13].

As mentioned above, there are three agents available, irinotecan, nivolumab and trifluridine/tipiracil, for the third- or later line treatment of HER2-negative AGC. Although the third- or later line treatment has tolerable treatment options for patients with AGC, their response rates have been shown to be 10% or less, with more than half of patients showing disease progression as the best response, leading to median progression-free survival (PFS) in as short as a couple of months [14]. In other words, treatment with these regimens can only aim at stable disease in the short term. Considering that few patients can receive fourth- or later line chemotherapy, it is very important to develop more active chemotherapy regimens in the third-line treatment.

Basically, oxaliplatin has a spectrum of activity and toxicity different from cisplatin, and shares a partial cross-resistance with cisplatin [15]. Tsuji *et al.* retrospectively reported the efficacy of modified fluorouracil, leucovorin and oxaliplatin (mFOLFOX6) as fourth- or later line treatment in 14 patients who were refractory or intolerant to fluoropyrimidines, cisplatin, taxanes and irinotecan: objective response rate (ORR) of 23.1%, median progression-free survival (PFS) of 3 months and MST of 8.9 months [16]. Similarly, in the retrospective analysis of 50 patients with similar medical history, Kondoh *et al.* reported that mFOLFOX6 provided ORR of 21.2%, time to treatment failure (TTF) of 2.4 months, and MST of 4.2 months [17]. Thus, oxaliplatin is often used in third- or later line treatment even for patients who have history of platinum administration, and an ORR around 20% has been reported consistently (Table 1) [16–21]. Thus, oxaliplatin can be a candidate agent to be included in the salvage-line chemotherapy for AGC.

**Table 1.** Results of studies using oxaliplatin-combination therapy after second-line chemotherapy.

	Tsuji *et al.* Retrospective study n = 14	Kondoh *et al.* Retrospective study n = 50	Kim *et al.* Phase II study n = 42	Kim *et al.* Phase II study n = 26	Suh *et al.* Phase II study n = 33	Seo *et al.* Retrospective study n = 62
Ref.	[16]	[17]	[18]	[19]	[20]	[21]
Line	4th–6th	4th	2nd–4th	2nd	2nd	2nd
Regimen	mFOLFOX6	SOX, CapeOX, mFOLFOX6	mFOLFOX6	mFOLFOX6	mFOLFOX6	mFOLFOX6
MST (months)	8.9	4.2	6.2	7.3	7.9	8.0
mPFS (months)	3.0	TTF 2.4	3.0	4.3	3.5	3.0
ORR%	23	21.2	21	26	27	22.6

CapeOX: Capecitabine plus oxaliplatin; mFOLFOX6: Modified fluorouracil, leucovorin and oxaliplatin; mPFS: Median progression-free survival; MST: Median survival time; ORR: Objective response rate; SOX: S-1 (tegafur–gimeracil–oteracil potassium) plus oxaliplatin; TTF: Time-to-treatment failure.

Neddylation is the process in which NEDD8 (neural precursor cell expressed developmentally down-regulated protein 8), an ubiquitin-like protein (UBL) with high similarity to ubiquitin, is conjugated to its target proteins. Activation of NEDD8 pathway, which is begun by the activation of NEDD8 activating enzyme (NAE), requires binding of NEDD8 to cullin-dependent E3 ubiquitin ligase. The complex of cullin-1, ROC1, Skp1 and F-box protein is called SCF complex and constitutes E3 ubiquitination enzyme. SCF complex is activated in several types of cancer [22,23], and cell growth is suppressed by inactivation of SCF complex [24–27]. In addition, it has been reported that inactivation of SCF complex causes accumulation of Cdc10-dependent transcript 1 (Cdt1). Cdt1 is essential for DNA replication, whose accumulation at the end of the cell cycle promotes the formation of pre-replicative complexes and replication in the next cell cycle. Overexpression of Cdt1, leads to overreplication and chromosomal instability in lower eukaryotes and recently in human cell lines [28]. Thus, NEDD8 pathway regulates to ubiquitinate substrate proteins that play a pivotal role in cell cycle progression and signal transduction at proper timing, and then these proteins are broken down by proteasome. Since these cellular processes are important for the growth and survival of tumor cells, inhibitors of NAE have a potential to be a target of cancer therapy by inhibiting proteasome-dependent breakdown of various proteins having pivotal biological function.

Pevonedistat (MLN4924, TAK-924) is a low-molecular weight first-in-class inhibitor of NAE. Pevonedistat has been initially developed as a therapeutic agent targeting hematologic malignancies. A preclinical study using a gastric cancer cell line also reported significant suppression of cell growth by pevonedistat [29]. Furthermore, while platinum agent causes DNA cross-liking, it induces protein complex to repair, which is known as resistant mechanism of oxaliplatin. However, as these proteins complex are broken down through neddylation, pevonedistat may conquer resistant mechanism [30].

From these findings, it is expected that the combination of pevonedistat and CapeOX exerts synergistic effect in patients with AGC who are refractory to platinum. This study is planned to assess the safety and tolerability of pevonedistat in combination with CapeOX as third-or later line treatment in patients with AGC who were previously treated with fluoropyrimidines (including oral agents) and a platinum containing drug (cisplatin or oxaliplatin) as first-line treatment.

## Study design

This trial is a multicenter, open-label, Phase I trial in Japanese patients with AGC refractory or intolerant to fluoropyrimidines, platinum containing drugs and taxane. This trial consists of the dose-finding part where the recommended dose is determined based on the incidence of dose-limiting toxicity (DLT), and the expansion part where the efficacy and safety of pevonedistat is assessed at the recommended dose (RD), associated with exploratory translational research.

## Objectives

The primary objectives of this study are to determine the recommended dose of pevonedistat in combination with CapeOX, and to examine the pharmacokinetics (PK) of pevonedistat in this combination.

The secondary objective is to assess the therapeutic effect of this combination chemotherapy in Japanese AGC patients who are refractory or intolerant to fluoropyrimidines, platinum containing drugs and taxanes.

The exploratory objectives are to explore the relationship of the molecular biologic profile of serum components such as micro-RNAs and proteins (proteins involved in carbohydrate or amino acid metabolisms) to the therapeutic effect of this combination chemotherapy.

## Eligibility criteria

Major inclusion criteria are as follows:

■ Aged 20 years old or older at the time of registration
■ ECOG performance status (PS) of 0 or 1
■ Adequate oral intake
■ Having histologically confirmed gastric or gastroesophageal junction adenocarcinoma
■ Prior two or more lines of chemotherapy, and refractory or intolerant to fluoropyrimidines (5-FU, capecitabine or S-1) and platinum (cisplatin or oxaliplatin) in the first-line chemotherapy and taxanes in the second-line chemotherapy
■ Having unresectable advanced or recurrent disease
■ Having at least one measurable lesion detected by thoracic-abdominal-pelvic CT scan (thickness: 0.5 mm or less)
■ No massive ascites reaching the upper abdomen over the pelvic cavity, and patients without massive ascites requiring drainage
■ No or grade 1 peripheral sensory neuropathy and peripheral motor neuropathy defined by CTCAE v5.0
■ No systemic antineoplastic therapy (including investigational drugs) within 14 days before the registration
■ No radiation therapy to their spinal cord or pelvis within 28 days before the registration, and no radiation therapy to the organs other than the spinal cord and pelvis within 14 days before the registration
■ Meeting the following criteria of clinical laboratory test within 3 days before the registration

- Aged 20 years old or older at the time of registration
- ECOG performance status (PS) of 0 or 1
- Adequate oral intake
- Having histologically confirmed gastric or gastroesophageal junction adenocarcinoma
- Prior two or more lines of chemotherapy, and refractory or intolerant to fluoropyrimidines (5-FU, capecitabine or S-1) and platinum (cisplatin or oxaliplatin) in the first-line chemotherapy and taxanes in the second-line chemotherapy
- Having unresectable advanced or recurrent disease
- Having at least one measurable lesion detected by thoracic-abdominal-pelvic CT scan (thickness: 0.5 mm or less)
- No massive ascites reaching the upper abdomen over the pelvic cavity, and patients without massive ascites requiring drainage
- No or grade 1 peripheral sensory neuropathy and peripheral motor neuropathy defined by CTCAE v5.0
- No systemic antineoplastic therapy (including investigational drugs) within 14 days before the registration
- No radiation therapy to their spinal cord or pelvis within 28 days before the registration, and no radiation therapy to the organs other than the spinal cord and pelvis within 14 days before the registration
- Meeting the following criteria of clinical laboratory test within 3 days before the registration
  - Neutrophil count ≥1500/mm^3^
  - Thrombocyte count ≥10 × 10^4^/mm^3^
  - Hemoglobin content ≥8.0 g/dl
  - AST (GOT) <100 U/l, ALT (GPT) <100 U/l
  - Total bilirubin ≤ upper limit of the normal range
  - Creatinine clearance ≥50 ml/min
- Provision of written informed consent to participate in the trial

Major exclusion criteria are as follows:

- Synchronous or metachronous (within 5 years) malignancies, except for carcinoma *in situ* or mucosal tumors curatively treated with local therapy
- Metastasis to the central nervous system (brain, spinal cord, meningeal) is evident
- Major surgery or laparotomized biopsy under general anesthesia within 14 days prior to registration
- Patients with an infectious disease requiring systemic treatment
- Intolerant to capecitabine and/or oxaliplatin
- Prothrombin time (PT) or activated partial thromboplastin time (APTT) exceeds 1.5-times of the upper limit of the institution standard value
- Left ventricular ejection fraction (LVEF) measured using echocardiography (ECHO), <50%
- Uncontrolled high blood pressure (i.e., systolic blood pressure >180 mmHg; diastolic blood pressure >95 mmHg)
- Prolonged rate corrected QT (QTc) interval ≥500 ms, calculated according to institutional guidelines
- Judged to be incapable of providing consent for certain reasons, such as concurrent dementia
- Any serious medical or psychiatric illness that could, in the investigator's opinion, potentially interfere with the completion of study procedures

## Planned treatment

**Figure 1.**
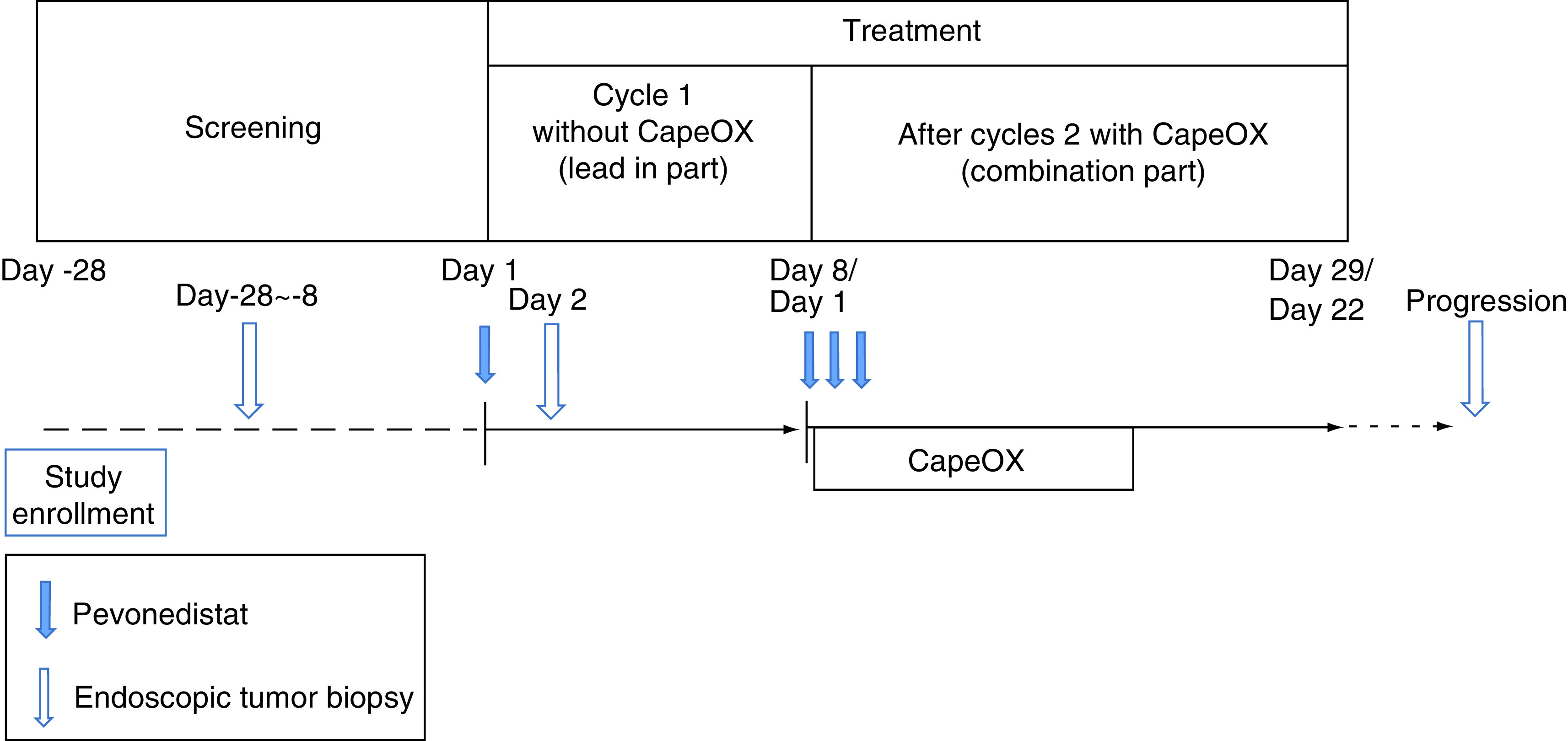
Treatment schedule. CapeOX: Capecitabine plus oxaliplatin.

**Figure 2.**
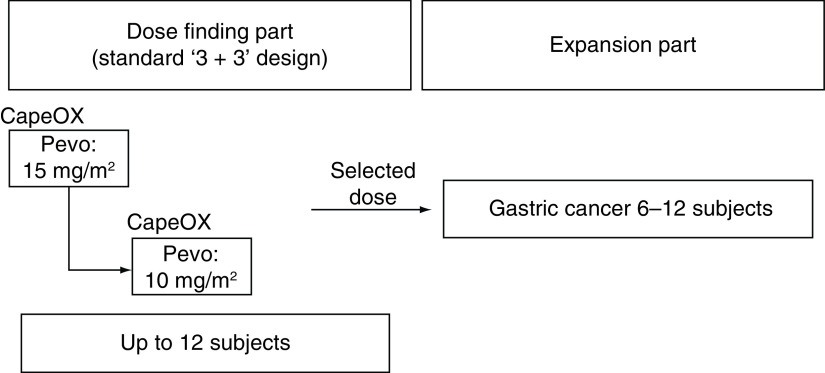
Dose de-escalation method. CapeOX: Capecitabine plus oxaliplatin; Pevo: Pevonedistat.

This trial consists of two parts: the dose-finding part where the recommended dose is determined based on the incidence of DLT at each dose level, and the expansion part where the efficacy and safety of this combination chemotherapy at the RD are assessed. In both cohorts, treatment schedule consists of lead-in part of monotherapy with pevonedistat alone for translational research followed by combination part (Figure 1 & Table 2), and the dose level in the dose-finding part is shown in the Figure 2.

**Table 2.** Treatment schedule.

	Lead-in		Cycle 1 (21 days)						Cycle 2 (21 days)	
Day	1	…	1 (day 8)	3	5	…	15	…	1 (day 29)	…
Pevonedistat	◯		◯	◯	◯				◯	
Oxaliplatin			◯						◯	
Capecitabine			◯ (from evening)	◯	◯	…	◯ (to morning)		◯	

### Lead-in part

Lead-in part is started within 7 days after the registration. Pevonedistat is administered only on day 1, and endoscopic biopsy for translational research is collected on day 2.

### Combination part

Cycle 1 of combination chemotherapy is started on the same day of the next week after the first administration of pevonedistat in the lead-in part. Oxaliplatin (130 mg/m^2^) is administered on day 1, capecitabine (1000 mg/m^2^/day, orally, twice daily with ~12-h interval) for 14 days, and pevonedistat is administered on days 1, 3 and 5 in a 3-week cycle, which is repeated until the patient meets the discontinuation criteria.

## Definition of DLT & RD

The DLT observation period is set from the initiation of the first administration in the lead-in part to days 35 or the day of the initiation of cycle 2, whichever comes earlier. However, an adverse event for which causality of the protocol treatment can be ruled out is not being regarded as DLT. RD is determined referring to the all available information about the adverse events observed in cycle 2 or later cycles in a comprehensive manner. The DLT is defined as follows:

- Grade 4 ‘decreased neutrophil count’ lasting for 7 days or longer
- Grade 3 ‘febrile neutropenia’ that requires dose delay
- Grade 4 thrombocytopenia lasting for 7 days or longer
- Grade 3 thrombocytopenia with bleeding
- Platelet count <10,000/mm^3^ at any time
- Grade 3 or greater PT or aPTT elevation in the absence of anticoagulation therapy
- Grade 2 or greater elevation of the PT or aPTT that is associated with clinically significant bleeding (CNS, GI, etc.)
- Grade 4 nonhematologic toxicity (except the following adverse events)
  Increased ALP, increased γ-GTP, hyperglycemia, hypercalcemia, hypocalcemia, hypernatremia, hyponatremia, hyperkalemia, hypokalemia, hypomagnesemia, hypophosphatemia, hypercholesterolemia, hypertriglyceridemia, Grade 4 nausea/vomiting/diarrhea that improves to grade ≤2 within 72 h
- Increased AST or increased ALT” (>5–20 × ULN) lasting for 8 days or longer
- Elevations of transaminases (>3–5 × ULN) and bilirubin (>1.5–3 × ULN) lasting for 3 days or longer between doses of pevonedistat
- In cases where pevonedistat cannot be administered in three doses by day 15 of the first cycle or administration of pevonedistat is discontinued due to an adverse event of which causal relationship with pevonedistat cannot be ruled out
- Dose delay of cycle 2 for 21 days or longer due to an adverse event of which causal relationship with pevonedistat cannot be ruled out
- Relative dose intensity of capecitabine in cycle 1 was less than 60% of its planned dose
- Discontinuation of the protocol treatment before second cycle due to adverse events other than (1)–(10) of which causal relationship with pevonedistat cannot be ruled out

## Planned sample size

The dose finding part is composed of the standard ‘3 + 3’ dose escalation design for which three to six subjects will be enrolled at each dose level depending on the occurrence of DLTs. In the dose level of pevonedistat at 20 mg/m^2^ (level 1) combined with CapeOX, two patients were previously enrolled. These two patients experienced a DLT of grade 2 or 3 elevation in ALT and AST in cycle 1, which led to delay in starting cycle 2. Due to the number of DLTs observed, no additional patients were enrolled in pevonedistat at 20 mg/m^2^ (level 1), and pevonedistat dose escalation to 25 mg/m^2^ (level 2) was stopped. Thereafter, the protocol was amended so that the dose levels of pevonedistat are: level 0, 15 mg/m^2^; level-1, 10 mg/m^2^.

After determination of the recommended dose in the dose finding part, subject will be enrolled into the expansion cohorts. The sample size of 10 in the expansion cohort is determined based on an analysis for futility in order to speed up therapeutic development. The first stage of Simon's two-stage design is used to calculate the probability of early termination when the null hypothesis is true, PET (*p*_0_), assuming the threshold response rate ≤10%. When the number of responses is ≤1/10, PET (*p*_0_) is 0.74. If the number of responses is ≥2/10, the subsequent study of this combination chemotherapy can be considered.

### Planned study period

2.5 years (scheduled from April 2019 to September 2021).

## Biomarker analysis

Exploratory objectives are to explore the relationship of the molecular biologic profile of serum components such as micro-RNAs and proteins (proteins involved in carbohydrate or amino acid metabolisms) with the therapeutic effects. In addition, items for analysis are gene and protein expressions induced by NAE inhibition using biopsied tumor tissue and serum samples in the lead-in part.

## Participating institutions

The participating institutions are as follows: Saitama Cancer Center, National Cancer Center Hospital, Cancer Institute Hospital of Japanese Foundation for Cancer Research.

## Conclusion

If this study demonstrates the safety of the combined administration of pevonedistat and CapeOX in patients with AGC, it may provide new treatment option to the patients. It is expected that the treatment improves overall prognosis of patients with AGC.

**Executive summary**

**Background**

- Pevonedistat (MLN4924, TAK-924) is a low-molecular weight first-in-class inhibitor of NEDD8 (neural precursor cell expressed developmentally down regulated protein 8) activating enzyme (NAE).
- It is expected that the combination of pevonedistat and CapeOX exerts synergistic effect in patients with gastric cancer who are refractory to platinum.

**Objective**

- The primary end points are to assess the safety and tolerability of the combination of pevonedistat and CapeOX in Japanese patients with unresectable advanced/recurrent gastric cancer who are refractory or intolerant to fluoropyrimidines, platinum containing drugs and taxanes and to examine the pharmacokinetics (PK) of pevonedistat under its combined administration with CapeOX.

**Patients & treatment**

- Patients with unresectable advanced/recurrent gastric cancer who are refractory or intolerant to fluoropyrimidines, platinum containing drugs and taxanes are enrolled, and receive pevonedistat, capecitabine and oxaliplatin.

**Trial design**

- This trial is a Phase I trial consisting of the dose finding part and the expansion part. The planned sample size is 20 in total.
